# Comparison of Health-Related Quality of Life Between Ileal Conduit Diversion and Orthotopic Neobladder in Women: A Meta-Analysis

**DOI:** 10.3389/fonc.2022.862884

**Published:** 2022-03-28

**Authors:** Wenzhou Xing, Sheng Zeng, Zhaoliang Xu, Shaoqiang Xing, Qian Liu

**Affiliations:** ^1^ Department of Urology, Tianjin First Central Hospital, Tianjin, China; ^2^ Department of Urology, First Teaching Hospital of Tianjin University of Traditional Chinese Medicine, Tianjin, China; ^3^ Department of Urology, Weihai Central Hospital, Shandong, China

**Keywords:** bladder cancer, health-related quality of life, orthotopic neobladder, ileal conduit diversion, meta-analysis

## Abstract

**Background:**

Orthotopic neobladder (ONB) reconstruction and ileal conduit diversion (ICD) can have different impacts on health-related quality of life (HRQOL) in patients with bladder cancer.

**Purpose:**

To conduct a meta-analysis to explore the comparison of HRQOL between ICD and ONB in women.

**Methods:**

PubMed, Embase, and the Cochrane Library were searched for available papers published from inception up to December 2020. The outcomes were the score data from HRQOL questionnaires. The random-effects model was used for all analyses.

**Results:**

Four studies (six datasets; 283 patients) were included. In the EORTC-QLQ-C30, there were no differences between ICD and ONB regarding cognitive functioning (weighted mean difference (WMD)=1.18, 95% confidence interval (CI): -20.52,22.88, P=0.915), global health (WMD=1.98, 95%CI: -15.26,19.22, P=0.822), emotional functioning (WMD=0.86, 95%CI: -19.62,21.33, P=0.935), physical functioning (WMD=0.94, 95%CI: -11.61,13.49, P=0.883), role functioning (WMD=-4.94, 95%CI: -12.15,2.27, P=0.180), and social functioning (WMD=-4.71, 95%CI: -20.83,11.40, P=0.567). There were no differences between ONB and ICD for specific symptoms (fatigue, nausea and vomiting, and pain) and single items (dyspnea, insomnia, appetite loss, constipation, diarrhea, and financial difficulties) (all P>0.05). In EORTC-QLQ-BLM30, there were no differences between ICD and ONB regarding bowel symptoms (WMD=5.45, 95%CI: -15.30,26.20, P=0.607), body image (WMD=-13.12, 95%CI: -31.15,4.92, P=0.154), sexual functioning (WMD=-5.55, 95%CI: -14.96,3.85, P=0.247), and urinary symptom (WMD=5.50, 95%CI: -7.34,18.34, P=0.401), but one study reported better future perspective with ONB (WMD=-14.9, 95%CI: -27.14,-2.66, P=0.017).

**Conclusion:**

Women who underwent ONB do not appear to have a statistically significantly better HRQOL than women who underwent ICD, based on EORTC-QLQ-C30 and EORTC-QLQ-BML30.

## Introduction

Bladder cancer ranks 10^th^ in worldwide cancer incidence. It is the 6^th^ most common cancer in men and the 17^th^ most common cancer in women, with an estimated 549,393 new cases and 199,922 deaths in 2018 (1). It mostly affects persons >55 years of age (2–4), it is 3.5 times more likely in men than in women (3–6), and it is about 3 times more likely in developed countries than in developing countries (7). It can be classified into non-muscle-invasive and muscle-invasive diseases based on invasion depth (4–7). The exact cause is unknown, but it is likely multifactorial and may include a combination of environmental factors, chronic bladder irritation, and genetic factors (5–7). The most important risk factors are tobacco smoke (active and passive) and professional exposure to carcinogens (5–7). Chronic bladder irritation from chronic urinary tract infection, more common in women than in men, is a risk factor for bladder cancer (5, 6). The management of bladder cancer is multidisciplinary (2, 4, 8, 9). The 5-year relative survival is 70% with localized disease, 35% with regional disease, and 5% with a distant-stage disease (10).

Radical cystectomy with urinary diversion is indicated for advanced disease (4, 9, 11). In women, anterior pelvic exenteration with urinary tract diversion is currently considered the standard of care in patients with non-metastatic muscle-invasive bladder cancer or non-muscle-invasive bladder cancer after failure of intravesical therapy (4–6). Although it is associated with satisfying midterm oncological outcomes, this invasive procedure is associated with a significantly decreased quality of life (QOL) (12–15). Urinary diversion is associated with a significant impact on sexual function, urinary continence, body image, and bowel function (6, 7).

Among urinary diversions options, orthotopic neobladder (ONB) reconstruction has been suggested as an interesting alternative to the classical ileal conduit diversion (ICD) (16–20). Indeed, such a reconstructive surgery is supposed to minimize the body image’s alteration while maintaining micturition through the urethra without increasing peri-operative complications. Still, it has been reported to expose the patient to specific long-term complications such as urinary incontinence or urinary retention requiring intermittent self-catheterization (16–21). Hence, whether ONB reconstruction is superior to ICD concerning health-related QOL (HRQOL) remains controversial. Shi et al. (22) performed a meta-analysis and demonstrated a significant difference favoring ONB patients in global health status, physical functioning, role functioning, and social functioning based on the European Organization for Research and Treatment of Cancer quality of life questionnaire (EORTC-QLQ-C30) (23–25). Nevertheless, that previous meta-analysis (22) did not specifically examine HRQOL in women.

Therefore, this systematic review and meta-analysis aimed to explore the comparison of HRQOL between ICD and ONB in women. The results could help determine which of the two methods would benefit the women the most in terms of HRQOL after bladder cancer surgery.

## Materials and Methods

### Literature Search

This systematic review and meta-analysis was performed according to the Preferred Reporting Items for Systematic Reviews and Meta-Analyses (PRISMA) guidelines (26). The PICO principle (27) was applied to define the research question and search for articles, followed by screening based on the eligibility criteria. PubMed, Embase, and the Cochrane Library were searched for available papers published from inception up to December 2020 using the MeSH terms of ‘Orthotopic neobladder’, ‘Ileal conduit diversion’, and ‘Health-related quality of life’, as well as relevant key words.

### Eligibility Criteria

The eligibility criteria were 1) population: women who underwent ONB or ICD; 2) exposure: ONB, 3) control: ICD, 4) outcome: HRQOL, and 5) language: English.

### Data Extraction

Study characteristics (authors, year of publication, country, study design, sample size, pathological stage classification, and follow-up duration) and outcome (score data from questionnaires) were extracted by two different investigators (Wenzhou Xing and Sheng Zeng) according to a pre-defined data extraction sheet that covered the data of interest. Discrepancies were resolved by discussion until a consensus was reached.

### Quality of the Evidence

The quality of the included studies and the risk of bias was assessed independently by two authors (Wenzhou Xing and Sheng Zeng) according to the Agency for Healthcare Research and Quality (AHRQ) methodology checklist for cross-sectional studies (28). Discrepancies in the assessment were resolved through discussion until a consensus was reached.

### Statistical Analysis

All analyses were performed using STATA SE 14.0 (StataCorp, College Station, Texas, USA). Weighted mean differences (WMDs) with 95% confidence intervals (CIs) were calculated for continuous data with significance denoted at P<0.05. Statistical heterogeneity among studies was calculated using Cochran’s Q-test and the I^2^ index. An I^2^ >50% and Q-test P<0.10 indicated high heterogeneity. The random-effects model was used for all analyses to account for differences among studies regarding patient populations, local practices, and changes over time (29–31). P-values <0.05 were considered statistically different. Possible publication bias was not examined by funnel plots and Egger’s test because the number of studies included in each quantitative analysis was <10, in which case the funnel plots and Egger’s test could yield misleading results (32, 33).

## Results

### Selection of the Studies

**Figure 1:**
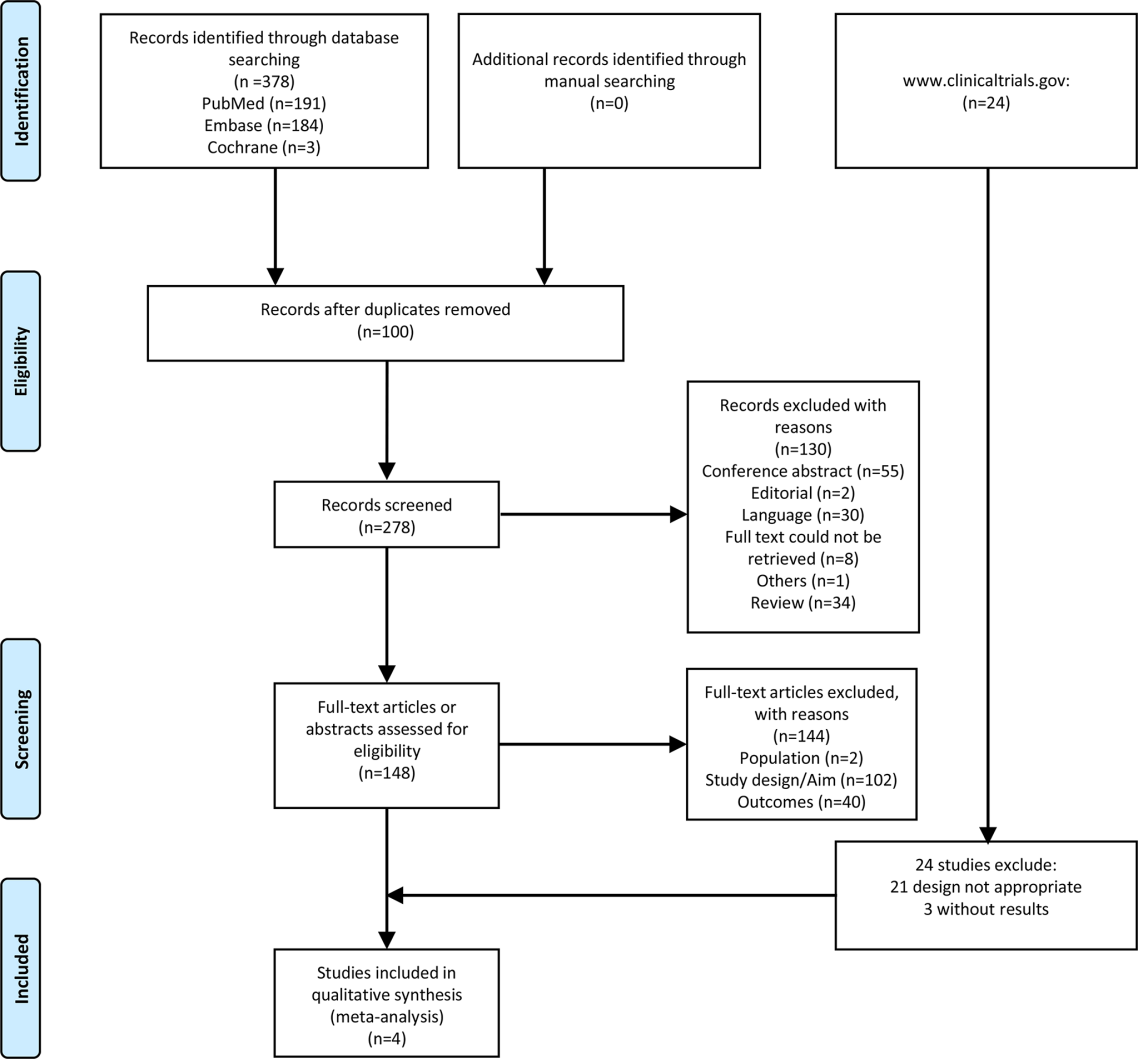
Flow diagram of the study selection process.

**Figure 1** presents the study selection process. The initial search yielded 378 records. After excluding 100 duplicates, 278 records were screened based on the titles, and 130 were excluded. Then, 148 full-text papers or abstracts were assessed for eligibility, and 144 were excluded (population, n=2; study design/aim, n=102; outcomes, n=40).

Finally, four studies (six datasets) were included (**Table 1**). There were 283 patients: 134 who underwent ONB and 149 who underwent ICD. Three studies (three datasets) were from Europe (34, 36, 37), and one study (three datasets) was from Egypt (35). The mean age of the patients was from 57.0 ± 10.3 to 71.8 ± 7.0 years. All four studies used the EORTC-QLQ-C30 (34–37), and three studies also used the EORTC-QLQ-BLM30 (34, 36, 37). On the AHRQ quality scale, three studies (three datasets) scored 11 points (34, 36, 37), and one study (three datasets) scored 10 points (35) (**Supplementary Table S1**).

**Table 1:** Literature search and characteristics of the included studies.

Study, year	Country	Design	No. of patients, n			Age, years, mean ± SD		Pathological stage classification				Follow-up, month, Mean ± SD		Outcome (Questionnaire)
								0-II		≥III				
			Total	ONB	ICD	ONB	ICD	ONB	ICD	ONB	ICD	ONB	ICD	
Gacci, 2013 (34)	Italy	Cross-sectional study	25	9	16	71.8 ± 7.0	74.4 ± 8.8	4	13	5	3	60.1 ± 21.5		EORTC-QLQ-C30, EORTC-QLQ-BLM-30
Zahran, 2017a (35)	Egypt	Cross-sectional study	43	22	21	61.4 ± 11.2	63.3 ± 5.7	/	/	/	/	/	/	EORTC-QLQ-C30
Zahran, 2017b (35)	Egypt	Cross-sectional study	47	27	20	57 ± 10.3	63.3 ± 5.7	/	/	/	/	/	/	EORTC-QLQ-C30
Zahran, 2017c (35)	Egypt	Cross-sectional study	55	35	20	61.8 ± 6.6	63.3 ± 5.7	/	/	/	/	/	/	EORTC-QLQ-C30
Siracusano, 2019 (36)	Italy	Cross-sectional study	73	24	49	67 ± 10.5	73 ± 8.6	15	29	9	20	43 ± 30.5	54 ± 36.8	EORTC-QLQ-C30, QLQ-BLM-30
Biardeau, 2020 (37)	France	Cross-sectional study	40	17	23	60.0 ± 7	71.0 ± 11.8	12	13	5	10	/	/	EORTC-QLQ-C30, EORTC-QLQ-BLM30
								

ICD, ileal conduit diversion; ONB, orthotopic neobladder.

### Comparison of ONB and ICD on HRQOL

**Figure 2:**
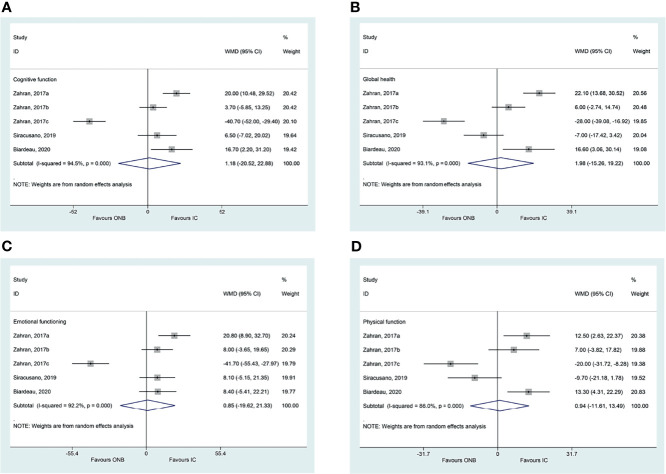
**(A)** Forest plot of cognitive functioning in women with bladder cancer who underwent ileal conduit diversion (IC) or orthotopic neobladder (ONB) **(B)** Forest plot of global health in women with bladder cancer who underwent ileal conduit diversion (IC) or orthotopic neobladder (ONB). **(C)** Forest plot of emotional functioning in women with bladder cancer who underwent ileal conduit diversion (IC) or orthotopic neobladder (ONB) **(D)** Forest plot of physical functioning in women with bladder cancer who underwent ileal conduit diversion (IC) or orthotopic neobladder (ONB).

**Figure 3:**
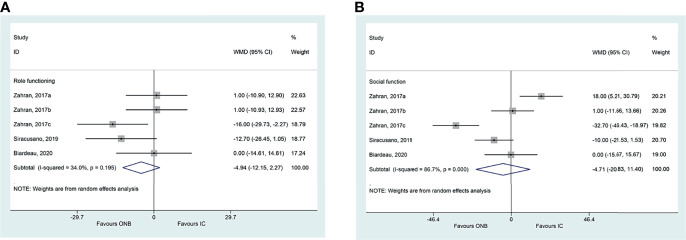
**(A)** Forest plot of role functioning in women with bladder cancer who underwent ileal conduit diversion (IC) or orthotopic neobladder (ONB). **(B)** Forest plot of social functioning in women with bladder cancer who underwent ileal conduit diversion (IC) or orthotopic neobladder (ONB).

There were no differences between ICD and ONB regarding cognitive functioning (WMD=1.18, 95%CI: -20.52,22.88, P=0.915; I^2^ = 94.5%, P_heterogeneity_<0.001) (**Figure 2A**), global health (WMD=1.98, 95%CI: -15.26,19.22, P=0.822; I^2^ = 93.1%, P_heterogeneity_<0.001) (**Figure 2B**), emotional functioning (WMD=0.86, 95%CI: -19.62,21.33, P=0.935; I^2^ = 92.2%, P_heterogeneity_<0.001) (**Figure 2C**), physical functioning (WMD=0.94, 95%CI: -11.61,13.49, P=0.883; I^2^ = 86.0%, P_heterogeneity_<0.001) (**Figure 2D**), role functioning (WMD=-4.94, 95%CI: -12.15,2.27, P=0.180; I^2^ = 34.0%, P_heterogeneity_=0.195) (**Figure 3A**), and social functioning (WMD=-4.71, 95%CI: -20.83,11.40, P=0.567; I^2^ = 86.7%, P_heterogeneity_<0.001) (**Figure 3B**). **Table 2** shows that there were no differences between ONB and ICD for all specific symptoms (fatigue, nausea and vomiting, and pain) and single items (dyspnea, insomnia, appetite loss, constipation, diarrhea, and financial difficulties) (all P>0.05).

**Table 2:** Summarized results of the meta-analysis by effects of orthotopic neobladder (ONB) versus ileal conduit diversion (ICD) on the HRQoL.

HRQoL questionnaire	Domains	Subdomains	No of studies	Total ONB patients	Total IC patients	WMD	95%CI	P	I^2^ (%)	P_heterogeneity_
EORTC-QLQ-C30	Global health		5	125	133	1.979	-15.261,19.219	0.822	93.1	<0.001
	Functioning Scale	Physical function	5	125	133	0.941	-11.609,13.492	0.883	86.0	<0.001
		Role functioning	5	125	133	-4.938	-12.149,2.274	0.180	34.0	0.195
		Emotional functioning	5	125	133	0.855	-19.624,21.333	0.935	92.2	<0.001
		Cognitive function	5	125	133	1.178	-20.524,22.879	0.915	94.5	<0.001
		Social function	5	125	133	-4.712	-20.829,11.404	0.567	86.7	<0.001
	Symptom scale	Fatigue	5	125	133	-10.221	-24.260,3.819	0.154	82.5	<0.001
		Nausea and vomiting	5	125	133	1.527	-2.961,6.014	0.505	0.0	0.844
		Pain	5	125	133	7.204	-4.621,19.029	0.232	78.8	0.001
	Single items	Dyspnea	5	125	133	5.779	-11.583,23.142	0.514	90.1	<0.001
		Insomnia	5	125	133	-7.474	-20.532,5.585	0.262	77.8	0.001
		Appetite loss	5	125	133	10.751	-5.518,27.020	0.195	88.1	<0.001
		Constipation	5	125	133	1.994	-4.471,8.459	0.546	0.0	0.993
		Diarrhea	5	125	133	1.330	-2.606,5.265	0.508	0.0	0.562
		Financial difficulties	5	125	133	8.949	-5.630,23.528	0.229	84.3	<0.001
EORTC-QLQ-BLM30	Urinary symptoms		1	17	23	5.500	-7.335,18.335	0.401		
	Bowel symptoms		2	41	72	5.448	-15.299,26.196	0.607	75.4	0.044
	Sexual functioning		2	41	72	-5.554	-14.959,3.852	0.247	42.6	0.187
	Body image		2	41	72	-13.116	-31.146,4.915	0.154	65.6	0.088
	Future perspective		1	24	49	-14.900	-27.144,-2.656	0.017		

**Figure 4:**
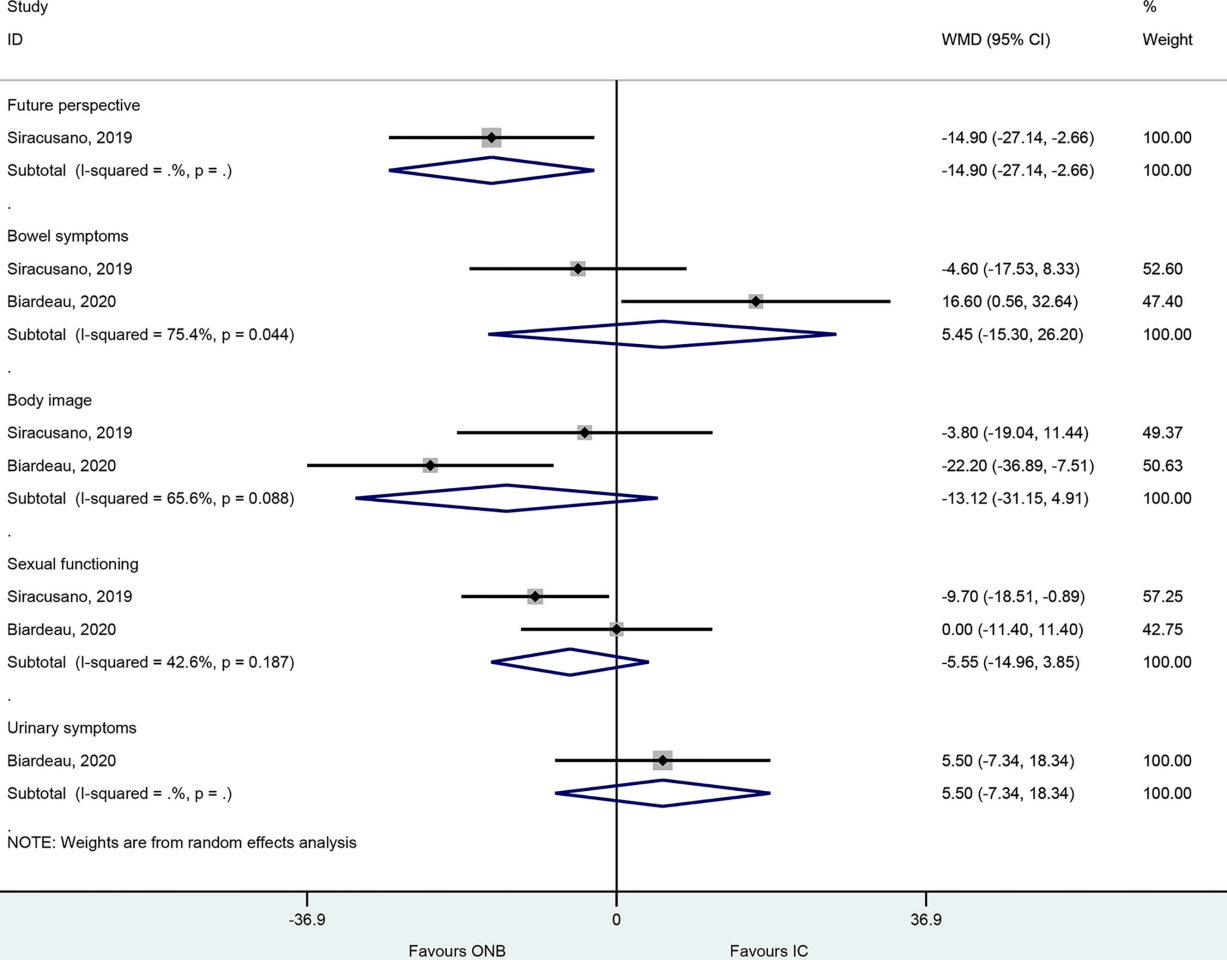
Forest plot of EORTC-QLQ-BLM30 in women with bladder cancer who underwent ileal conduit diversion (IC) or orthotopic neobladder (ONB).

In the EORTC-QLQ-BLM30, there were no differences between ICD and ONB regarding bowel symptoms (WMD=5.45, 95%CI: -15.30,26.20, P=0.607; I^2^ = 75.4%, P_heterogeneity_=0.44), body image (WMD=-13.12, 95%CI: -31.15,4.92, P=0.154; I^2^ = 65.6%, P_heterogeneity_=0.088), sexual functioning (WMD=-5.55, 95%CI: -14.96,3.85, P=0.247; I^2^ = 42.6%, P_heterogeneity_=0.187), and urinary symptom (WMD=5.50, 95%CI: -7.34,18.34, P=0.401) (**Figure 4**), but one study reported better future perspective with ONB (WMD=-14.9, 95%CI: -27.14,-2.66, P=0.017) (**Figure 4**).

## Discussion

The impact of ONB reconstruction vs. ICD on HRQOL in women with bladder cancer is poorly known. Therefore, this meta-analysis aimed to explore the comparison of HRQOL between ICD and ONB in women. The results showed that the patients who underwent ONB do not appear to have a statistically significantly better HRQOL than patients who underwent ICD, based on EORTC-QLQ-C30 and EORTC-QLQ-BML30.

HRQOL is a comprehensive and complex outcome that defines an individual’s subjective satisfaction after treatments for a specific condition (38). The EORTC-QLQ-C30 is a classical and well-known validated and reliable questionnaire used to assess cancer patients’ quality of life (23–25). It is translated into >100 languages and used in >5000 studies (qol.eortc.org). On the other hand, the EORTC-QLQ-BLM30 is a non-validated survey that examines 30 HRQOL items for patients with T2-T4 muscle-invasive bladder cancer (39). These two tools are widely used in patients with bladder cancer (39).

A previous meta-analysis by Shi et al. (22) reported that patients who underwent ONB were more likely to have a better HRQOL than those who underwent ICD, but patients with ONB were more likely to have urinary symptoms than those with ICD, specifically regarding global health status, physical functioning, role functioning, and social functioning. That previous meta-analysis included 26 studies and 2507 patients, irrespective of sex. Reviews by Cerruto et al. (40, 41) reported better global health status, physical functioning, role functioning, social functioning, cognitive functioning, and emotional functioning with ONB than with ICD. Given the difference in urological and sexual anatomy between males and females, there is a possibility that the outcomes of ONB and ICD might be different in females. Indeed, Cerruto et al. (41) indicated that sex was one factor that affected HRQOL after ONB or ICD. Females after radical cystectomy show worse emotional and role functioning, fatigue, and appetite (42, 43). In the present meta-analysis, there were no differences in any of the EORTC-QLQ-C30 items between ONB and ICD in women, and only one EORTC-QLQ-BLM30 item was favoring ONB, but it was based on only one study. Therefore, it could be hypothesized that the HRQOL benefits observed with ONB compared with ICD in the general bladder cancer patient population might be influenced by the males since they represent the majority of bladder cancer patients (1, 4–6, 10). Still, because of the eligibility criteria, only a few studies could be included in the present meta-analysis, far fewer than in previous reviews and meta-analyses (22, 40, 41, 43). Additional studies directly comparing the HRQOL in males and females after ONB and ICD are required. Currently, level 1 evidence favoring either ONB reconstruction vs. ICD in women is lacking.

Still, the present meta-analysis included four studies in women. All four studies were cross-sectional studies. The study by Gacci et al. (34) included 37 Italian women who underwent radical cystectomy and urinary diversion over a 9-year period. They reported that women who underwent ICD reported worse HRQOL than those who underwent ONB reconstruction (34). Siracusano et al. (36) included 73 women who underwent radical cystectomy and urinary diversion over a 7-year period at six hospitals in Italy. They reported that financial difficulties were the only difference between women who underwent ONB and ICD (36). Biardeau et al. (37) reported 40 women who underwent bladder cancer surgery and urinary diversion over 11 years at three hospitals in France. They reported no differences in HRQOL between women who underwent ONB or ICD. The study by Zahran et al. (35) included 145 Egyptian women, grouped in three subgroups/datasets according to the incontinence after ONB (total continence, nocturnal incontinence, and chronic urinary retention and maintained on clean intermittent catheterization). They concluded that ONB achieved better HRQOL than ICD but only if continence could be preserved; for women in whom incontinence could be expected, ICD was a better option than ONB (35). Hence, future studies should specifically examine the aspect of incontinence in women who undergo ONB or ICD after bladder cancer.

The present meta-analysis has limitations. All the included studies were cross-sectional, with inherent selection bias and differences in the demographic characteristics of the patients. Currently, there are no available preoperative data regarding HRQOL or longitudinal data collected at different intervals. Other factors, such as fatigue, nausea and vomiting, insomnia, and appetite loss, could not be fully analyzed. Heterogeneity was high in several analyses. Finally, because this meta-analysis only included studies in women, the sample size was small. An issue was that studies on HRQOL after bladder cancer and ONB/ICD reported their data for all patients without differences between males and females. Because less than 10 studies were included, publication bias could not be evaluated because such analysis could yield improper results (33).

## Conclusion

Women who underwent ONB do not appear to have a statistically significantly better HRQOL than women who underwent ICD, based on EORTC-QLQ-C30 and EORTC-QLQ-BML30. We have no evidence that women who underwent anterior pelvic exenteration for bladder cancer fared better after ONB than ICD in terms of HRQOL. Further prospective studies are needed to validate the findings.

## Data Availability Statement

The original contributions presented in the study are included in the article/**Supplementary Material**. Further inquiries can be directed to the corresponding author.

## Author Contributions

WZX: Project development, Data Collection, Data analysis, Manuscript writing, Manuscript editing; SZ: Data collection, Data analysis; ZLX: Manuscript editing; SQX: Manuscript editing; QL: Project development, Manuscript editing. All authors contributed to manuscript revision, read, and approved the submitted version.

## Funding

This study was funded by the National Natural Science Foundation of China (No. 51673150) and Tianjin Key Medical Discipline (Specialty) Construction Project.

## Conflict of Interest

The authors declare that the research was conducted in the absence of any commercial or financial relationships that could be construed as a potential conflict of interest.

## Publisher’s Note

All claims expressed in this article are solely those of the authors and do not necessarily represent those of their affiliated organizations, or those of the publisher, the editors and the reviewers. Any product that may be evaluated in this article, or claim that may be made by its manufacturer, is not guaranteed or endorsed by the publisher.
