# Supplementary material for: Comparison of Health-Related Quality of Life Between Ileal Conduit Diversion and Orthotopic Neobladder in Women: A Meta-Analysis
Source: Front Oncol. 2022 Mar 28;12:862884. doi: 10.3389/fonc.2022.862884 (PMC8996170; doi:10.3389/fonc.2022.862884)
Supplement: Supplementary file 1 [file Table_1.docx]

**Supplementary Table S1**. AHRQ quality assessment for cross-sectional studies

| Study | Define the source of information | List inclusion and exclusion criteria for exposed and unexposed subjects or refer to previous publications | Indicate time period used for identifying patients | Indicate whether or not subjects were consecutive if not population-based | Indicate if evaluators of subjective components of the study were masked to other aspects of the status of the participants | Describe any assessments undertaken for quality assurance purposes | Explain any patient exclusions from the analysis | Describe how confounding was assessed and/or controlled | If applicable, explain how missing data were handled in the analysis | Summarize patient response rates and completeness of data collection | Clarify what follow-up, if any, was expected and the percentage of patients for which incomplete data or follow-up was obtained | Total quality scores |
| --- | --- | --- | --- | --- | --- | --- | --- | --- | --- | --- | --- | --- |
| Gacci, 2013 (32) | ★ | ★ | ★ | ★ | ★ | ★ | ★ | ★ | ★ | ★ | ★ | 11 |
| Zahran, 2017a (35) | ★ | ★ | ★ | ★ | ★ | ★ | / | ★ | ★ | ★ | ★ | 10 |
| Zahran, 2017b (35) | ★ | ★ | ★ | ★ | ★ | ★ | / | ★ | ★ | ★ | ★ | 10 |
| Zahran, 2017c (35) | ★ | ★ | ★ | ★ | ★ | ★ | / | ★ | ★ | ★ | ★ | 10 |
| Siracusano, 2019 (33) | ★ | ★ | ★ | ★ | ★ | ★ | ★ | ★ | ★ | ★ | ★ | 11 |
| Biardeau, 2020 (34) | ★ | ★ | ★ | ★ | ★ | ★ | ★ | ★ | ★ | ★ | ★ | 11 |
